# Multimechanism Synergistic Photodetectors with Ultrabroad Spectrum Response from 375 nm to 10 µm

**DOI:** 10.1002/advs.201901050

**Published:** 2019-06-04

**Authors:** Xudong Wang, Hong Shen, Yan Chen, Guangjian Wu, Peng Wang, Hui Xia, Tie Lin, Peng Zhou, Weida Hu, Xiangjian Meng, Junhao Chu, Jianlu Wang

**Affiliations:** ^1^ State Key Laboratory of Infrared Physics Shanghai Institute of Technical Physics Chinese Academy of Sciences 500 Yu Tian Road Shanghai 200083 China; ^2^ University of Chinese Academy of Sciences 19 Yuquan Road Beijing 100049 China; ^3^ Department of Microelectronics State Key Laboratory of ASIC and System Fudan University Shanghai 200433 China

**Keywords:** 2D materials, ferroelectric, infrared detectors, pyroelectric, ultrabroad spectrum response

## Abstract

Broadening the spectral range of photodetectors is an essential topic in photonics. Traditional photodetectors are widely used; however, the realization of ultrabroad spectrum photodetectors remains a challenge. Here, a photodetector constructed by a hybrid quasi‐freestanding structure of organic ferroelectric poly(vinylidene fluoride–trifluoroethylene) (P(VDF‐TrFE)) with molybdenum disulfide (MoS_2_) is demonstrated. The 2D MoS_2_ with the ultrathin structure brings a great benefit of heat dissipation for the pyroelectric infrared detector. By coupling the mechanisms of pyroelectrics, photoconductor, and phototransistor effect, an ultrabroad spectrum response ranging from ultraviolet (375 nm) to long‐wavelength infrared (10 µm) is achieved. In the 2.76–10 µm spectral range, the 2D MoS_2_ is used to read and amplify the photocurrent induced by the pyroelectric effect of P(VDF‐TrFE). The sensitivity of the device in this spectral range is greatly enhanced. A high responsivity of 140 mA W^−1^, an on/off photocurrent switching ratio up to 10^3^, and a quick response of 5.5 ms are achieved. Moreover, the ferroelectric polarization field dramatically enhances the photoconductive properties of MoS_2_ and restrains dark current and noise. This approach constitutes a reliable route toward realizing high‐performance photodetectors with a remarkable ultrabroad spectrum response, high responsivity, low power consumption, and room‐temperature operation.

Photodetectors extract information from light and thus are critical optoelectronic components. The materials and structures of the photodetector are constantly updated. However, a photodetector with ultrabroad spectrum photoresponse, especially covering the ultraviolet (UV)–visible–short‐wavelength infrared (SIR)–mid‐wavelength infrared (MIR)–long‐wavelength infrared (LIR) range, is still a big challenge. Traditional semiconductors with narrow bandgaps, such as mercury cadmium telluride (HgCdTe), indium antimonide (InSb), and indium gallium arsenide (InGaAs), are generally employed in infrared detectors,^1^, ^2^, ^3^ but they typically must be operated at cryogenic temperatures to achieve high sensitivity. Thermal infrared detectors, such as pyroelectric detectors and thermistor bolometers, represent another prominent class of detectors for infrared detection.^4^, ^5^, ^6^, ^7^ Thermistor bolometers operate in DC mode and cause large power consumption, while pyroelectric detectors operating in AC mode have greater potential for large‐scale and low‐power‐consumption applications. Ferroelectrics, such as lithium tantalate (LiTaO_3_), lead zirconate titanate (PZT), and poly(vinylidene fluoride–trifluoroethylene) (P(VDF‐TrFE)), are usually used as the core materials for pyroelectric infrared sensors because of their excellent pyroelectric effect.^8^, ^9^, ^10^, ^11^, ^12^, ^13^ Pyroelectric photodetectors operate at room temperature; the cryogenic refrigeration can be avoided, which leads to a significant value in commercial applications. However, the performance bottleneck of traditional pyroelectric photodetectors is introduced by their shortcomings, such as imperfect thermal isolation structure, complex photodetector cells, small signal‐to‐noise ratio and limited sensitivity.

2D materials for photodetector fabrication have attracted extensive attention over the past decade.^14^, ^15^ Transition‐metal dichalcogenides (TMDs) constitute an important class of 2D materials; they have immense potential for future photodetector applications because of their excellent optoelectronic performance.^16^, ^17^, ^18^ Devices based on MoS_2_, a typical member of the TMDs family, exhibit ultrasensitive and ultrahigh gain characteristics for photodetector applications.^19^ However, the bandgap of MoS_2_, which ranges from 1.2 eV for bulk to 1.8 eV for single layer, results in a limited spectral response that terminates at SIR. 2D semiconductors with narrow bandgaps, such as platinum diselenide (PtSe_2_), black phosphorene, and black arsenic phosphorus, were recently discovered and used to fabricate the infrared detectors.^17^, ^20^, ^21^, ^22^, ^23^ Limited by absorption efficiency, these infrared detectors typically have low photocurrent gain and low quantum efficiency.

In this paper, we present a photodetector with ultrabroad spectrum response that constructed by a quasi‐freestanding hybrid structure of organic ferroelectrics with 2D semiconductor. The photoresponse range covers an ultrabroad spectrum from UV to LIR regions. This superior optoelectronic performance originates from the coupling of multimechanism in ferroelectric materials and 2D semiconductors, such as pyroelectricity, ferroelectricity, and photoconductivity, into a single device. The most impressive characteristic of this type of detector is that these mechanisms are synergistic. As one of the most promising 2D semiconductors, MoS_2_ has not only excellent semiconducting properties, but also limited thermal capacity in 2D regime. It is used to determine the variation in charge induced by infrared irradiation, which greatly improves sensitivity to the pyroelectric effect on ferroelectrics. Moreover, the ferroelectric polarization field is capable of not only restraining dark current in the full spectrum but also narrowing the bandgap of MoS_2_, which dramatically enhances the photoconductive properties of MoS_2_.

**Figure** **1**a illustrates the preparation process of the photodetector. In brief, for device fabrication, an ultrathin (1.7 µm) polyimide film was fabricated on the SiO_2_/Si substrate, and few‐layer MoS_2_ flakes were then transferred to this polyimide film. The chromium/gold (Cr/Au) source and drain contacts were prepared using UV lithography, metal thermal evaporator, and lift‐off steps. Subsequently, 300 nm P(VDF‐TrFE) was spin‐coated on MoS_2_ as the gate dielectric, and the patterned aluminum (Al) served as the gate electrode. Finally, the device with the polyimide film was delaminated from the SiO_2_/Si substrate. After that, the photodetector was only supported by the ultrathin polyimide substrate. More details are provided in the “Experimental Section.” Related optical images are displayed in Figure S1 (Supporting Information). Figure 1b presents a schematic of the device with incident light irradiation, where the basic structure of the photodetector combines MoS_2_ with P(VDF‐TrFE) to form a transistor. Figure 1c presents a diagram of a P(VDF‐TrFE) capacitor, which served as the functional material in the MIR–LIR range. With the irradiation of incident light in the MIR–LIR range, the charge on both sides of P(VDF‐TrFE) changed (Δ*Q*) with the temperature (Δ*T*),^11^ resulting in variations in the charge of the MoS_2_ channel in this device. Figure 1d displays the band structure schematic of MoS_2_, which is the functional material in the UV–SIR range.^24^ Based on the photoconductive effect of MoS_2_, lots of photogenerated carriers contribute to the photocurrent under UV–SIR incident light.^19^, ^25^

**Figure 1:**
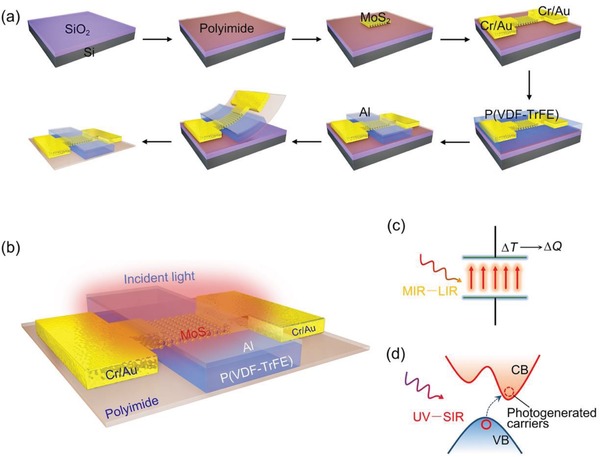
a) Schema illustrating the preparation process for the photodetector. b) 3D schematic of the photodetector located on an ultrathin (1.7 µm) polyimide substrate with incident light. c) Structural diagram of the polarized P(VDF‐TrFE). The temperature of P(VDF‐TrFE) changes (Δ*T* ) with the irradiation of light in the MIR–LIR range, resulting in changes in charge (Δ*Q*) on both sides of P(VDF‐TrFE). d) MoS_2_ band structure diagram, in which CB represents the conduction band and VB represents the valence band. With the illumination of UV–SIR light, the photogenerated carriers are excited from VB to CB, thus contributing to the photocurrent of the device.

The electronic properties of the device are presented in Figure S2 (Supporting Information). With the gate voltage (*V*_G_) sweeping at a fixed drain bias (*V*_SD_), the drain current (*I*_SD_) is found to show a large counterclockwise hysteresis window for the transfer curve, which is dominated entirely by the ferroelectric polarization effect of P(VDF‐TrFE).^26^ The ferroelectricity of P(VDF‐TrFE) is essential for the optoelectronic performance of the photodetector; so we characterized it according to the typical hysteresis loop displayed in Figure S2c (Supporting Information). The coercive voltage is ≈+26 or −20 V, where the asymmetric coercive voltage is primarily caused by the asymmetry of the upper and lower electrodes on P(VDF‐TrFE).^27^ When a negative voltage less than −20 V is applied, the molecular chains of P(VDF‐TrFE) are neatly arranged and polarized upward. After the removal of the external voltage, an upward remanent polarization field formed in P(VDF‐TrFE). With the modulation of these two polarization states, the MoS_2_ channel can be maintained in two stable states, which are typically used for nonvolatile memory applications.^26^, ^28^ In this study, we select the upward polarization state as the working mode for the photodetector (Figure S2a, Supporting Information)). After P(VDF‐TrFE) is polarized by the application of a sufficiently negative voltage to the gate, the orientation of the polarization can be maintained in the upward direction for an extended period without applying a gate voltage. Under these circumstances, any signal induced by the incident light can be interpreted according to the current variation in the MoS_2_ channel with a small drain bias.

**Figure** **2**a,b illustrates the working principle of the photodetector responding to incident light with a wavelength in the MIR–LIR range. In this spectral range, the functional material is P(VDF‐TrFE), and the auxiliary material is MoS_2_. Polarized P(VDF‐TrFE) is well known to exhibit excellent pyroelectric characteristics.^29^ We also prepared a P(VDF‐TrFE) capacitor located on silicon substrate; its pyroelectric current with periodic infrared irradiation is illustrated in Figure S3 (Supporting Information). Moreover, the temperature dependence of remanent polarization of P(VDF‐TrFE) is shown in Figure S4f (Supporting Information). In Figure 2a, the device is in thermal equilibrium without infrared irradiation. MoS_2_ is in a depleted state under the modulation of the remanent polarization field. Holes in MoS_2_ compensate for the remanent polarization field, which is bound at the interface between MoS_2_ and P(VDF‐TrFE). When the infrared light is irradiated on the device, as illustrated in Figure 2b, the remanent polarization for P(VDF‐TrFE) changes (*P* − ∆*P*) with the variation in temperature (*T* + ∆*T*). From the comparison of Figure 2b with Figure 2a, the polarization intensity decreases as the temperature of P(VDF‐TrFE) increases. Consequently, the number of holes in MoS_2_ bound by the polarized electric field reduces (elevates Fermi level), resulting in a multitude of electrons flowing through the channel. Thus, the drain current will increase (*I*_SD_ + ∆*I*_SD_) following infrared irradiation. Although the photodetector exhibits a substantial photoresponse for MIR and LIR radiation, which mainly depends on the pyroelectric effect, the underlying mechanism is entirely different from those of traditional pyroelectric detectors. As indicated in Figure 2c, a traditional pyroelectric detector unit contains numerous electronic components (e.g., a pyroelectric sensor, a capacitor, several resistors, and a metal‐oxide–semiconductor field effect transistor (FET)) used to record the photoresponse signal generated by infrared light irradiation. However, in our photodetector, such extra electronic components and complex readout circuits can be simplified into a single ferroelectric transistor, combining the advantages of MoS_2_ and P(VDF‐TrFE), to realize a high‐performance infrared photodetector. Moreover, the device only required a small drain bias during operation. Therefore, it is desirable for future ultralow‐power‐consumption applications.

**Figure 2:**
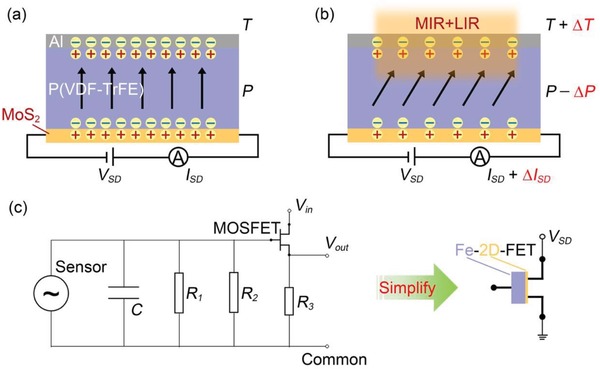
a) Cross‐sectional schematic of the Al‐P(VDF‐TrFE)‐MoS_2_ part in the device, displaying the electrical connections used to characterize the optoelectronic properties. P(VDF‐TrFE) is in an upward polarization state, and a small drain bias is applied to measure the current variation in the MoS_2_ channel. b) The photodetector responses to incident light wavelengths ranging from MIR to LIR. The polarization intensity of P(VDF‐TrFE) decreases from that in panel (a) following infrared irradiation because of the pyroelectric effect. c) In this study, the traditional pyroelectric detector unit is simplified into a single 2D semiconductor‐based FET gated by ferroelectric (Fe‐2D‐FET).

The photocurrent switching characteristics of the device with and without laser irradiation at wavelengths of 4 and 10 µm are shown in **Figure** **3**a and Figure 3c, respectively. The photocurrent switching ratio exceeding 1 × 10^3^ and 5 × 10^3^ is achieved after 4 and 10 µm laser irradiation, respectively. Infrared energy is much lower than the bandgap energy of MoS_2_ which cannot produce photogenerated carriers through the photoconductive effect, even though the bandgap of MoS_2_ can be narrowed to an extent by the polarization field.^30^ Such a substantial current increase in the MoS_2_ channel is certainly caused by the pyroelectric effect of P(VDF‐TrFE) under infrared irradiation. On one hand, when infrared radiation is incident onto the device, the local temperature increases, which causes the remanent polarization to decrease as a result of the pyroelectric effect in P(VDF‐TrFE). After the remanent polarization decreases, the Fermi level for MoS_2_ is elevated, which increases the number of electron carriers in the channel, eventually leading to an increment in drain current. On the other hand, without infrared radiation, the holes in MoS_2_ are bound by the upward remanent polarization field, leading to an ultralow dark current. Consequently, high on/off photocurrent switching ratios are achieved.

**Figure 3:**
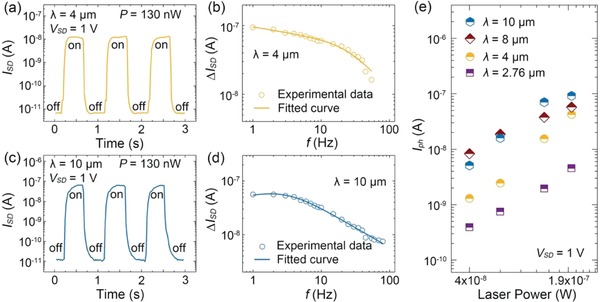
a) Photocurrent switching behavior of the device with MIR light (λ = 4 µm, *P* = 130 nW) at *V*_SD_ = 1 V. b) The photocurrent response (∆*I*_SD_) dependence of the photodetector as a function of frequency (  *f*  ) under 4 µm infrared light. The solid curve is the photoresponse current fitted by a phenomenological model. c) Photocurrent switching behavior of the photodetector for LIR light (λ = 10 µm, *P* = 130 nW) at *V*_SD_ = 1 V. d) Photocurrent response (∆*I*_SD_) dependence of the photodetector as a function of frequency (  *f*  ) under 10 µm infrared light. The solid curve is the photoresponse current fitted by a phenomenological model. e) Photocurrent dependence on incident light power of infrared light at wavelengths of 2.76, 4, 8, and 10 µm.

To characterize the pyroelectric response time of the photodetector, frequency‐dependent measurements of the photocurrent increment ∆*I*_SD_ are performed, the results are provided in Figure 3b,d. ∆*I*_SD_ is converted into a voltage signal by a preamp and recorded by a lock‐in amplifier. Similar to the voltage response of conventional pyroelectric detectors, the photocurrent response of the photodetector also decays with increasing frequency.^4^ Therefore, the photoresponse current *R_I_*(ω) can be accounted for by a phenomenological model and approximated as

(1) $$R_{I}(\omega )=F(\omega )/\surd (1+\tau^{2}\omega^{2})$$

where *F*(ω) is a rational polynomial term primarily related to the thermal propagation characteristics of P(VDF‐TrFE); the term 1/√(1 +τ^2^ω^2^) denotes the typical behavior of pyroelectric detectors, ω is the modulation frequency, and*τ* is the response time constant. The fitting curves based on Equation (1) (Figure 3b,d) reveal adequate agreement with experimental data; moreover, we obtain τ_1_ = 5.5 ms for 4 µm irradiation and τ_2_ = 52.5 ms for 10 µm irradiation. These fast photoresponse times can be attributed to the following two improvements. First, during the device fabrication, the polyimide substrate was thinned to 1.7 µm, thus producing a quasi‐freestanding P(VDF‐TrFE) film, and effectively reducing its heat capacitance.^31^, ^32^ The value of τ is mainly determined by the thermal behavior of P(VDF‐TrFE). More heat is distributed on P(VDF‐TrFE) when the substrate is thinner, resulting in higher sensitivity to the pyroelectric effect. Second, MoS_2_ in the 2D regime is ultrasensitive to interface characteristics, including traps, surrounding circumstance, and surface electric field.^33^, ^34^ In this device, the carrier concentration in MoS_2_ is completely modulated by the remanent polarization field. Therefore, the drain current is expected to demonstrate a fast photoresponse to variation in the remanent polarization field induced by infrared irradiation. Figure 3e illustrates the photocurrent dependence on laser power with different incident light wavelengths in the 2.76–10 µm range. The photocurrent increases exponentially with incident light power at *V*_SD_ = 1 V. To sum up, the photodetector embraces excellent optoelectronic performance under 2.76–10 µm irradiation as a result of the MoS_2_‐assisted pyroelectric effect in P(VDF‐TrFE). Figure S5a–c (Supporting Information) presents additional experimental data on photocurrent switching characteristics at other wavelengths in the MIR–LIR region. When P(VDF‐TrFE) is in the downward polarization state, a negative optoelectronic effect is observed with MIR irradiation, as shown in Figure S5d–f (Supporting Information). This phenomenon further confirms that the device photoresponse in this region originates from the MoS_2_‐assisted pyroelectric effect in P(VDF‐TrFE).

To analyze the noise source of the device, we measured the noise power density (NPD) in dark current (shown in **Figure** **4**a). The results demonstrate that the 1/*f* noise is the main noise source in this device, which originates from fluctuations in local electronic states induced by defects or disorders that commonly exist in devices based on 2D material‐based devices.^35^, ^36^ Figure 4b illustrates the responsivity (*R*) and on/off photocurrent switching ratio (on/off ratio) for incident light with different wavelengths in the 2.76–10 µm range. The photoresponsivity is calculated as *R* = *I*_ph_/*P*, where *I*_ph_ is the photocurrent and *P* is the power of incident light. In the 2.76–10 µm range, the highest responsivity of 140 mA W^−1^ with 10 µm incident light is achieved. High on/off photocurrent switching ratios around 10^3^ benefit from the sensitivity of MoS_2_ to the polarization electric field. The nonlinear tendency in Figure 4b is mainly introduced by the difference in the absorption efficiency of P(VDF‐TrFE).^11^, ^37^ Therefore, as the infrared detector, our device embraces advantages in terms of high sensitivity, high photocurrent gain, and fast response speed, thereby demonstrating its potential for application in high‐performance room‐temperature infrared detection.

**Figure 4:**
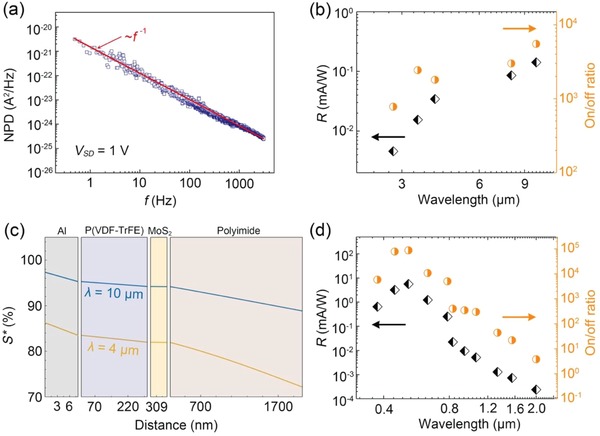
a) NPD varied with frequency (*f*) and was measured at room temperature, *V*_SD_ = 1 V was set according to the working conditions of the device. b) Photoresponsivity and on/off photocurrent switching ratio of the device with incident light of different wavelengths in the 2.76–10 µm range; *V*_SD_ = 1 V. c) Normalized energy flux (S*) profile for the device from top to bottom under infrared irradiation (λ = 4 µm and λ = 10 µm). d) Photoresponsivity and on/off photocurrent switching ratio of the photodetector for incident light wavelengths ranging from 375 nm to 2 µm at *V*_SD_ = 1 V.

The thermal profile is a critical factor affecting the pyroelectric effect of P(VDF‐TrFE). We calculate the thermal profile of the device from the top to the bottom under infrared irradiation, as shown in Figure 4c. Such a calculation provides a guidance for further optimization of device optoelectronic performance in infrared wavebands. In this calculation, from the top gate Al electrode to the bottom polyimide substrate, most of the energy is neither reflected nor transmitted but is rather absorbed by the polyimide substrate. A more detailed calculation is described in Note S1 (Supporting Information). The thermal profile is positively correlated with the material thickness, suggesting that improving the optoelectronic performance of the device requires the polyimide thickness to be further reduced and the P(VDF‐TrFE) thickness to be increased. Consequently, more incident infrared radiation energy can be absorbed by P(VDF‐TrFE). Moreover, this approach also effectively increases light transmission to the gate electrode, thereby preventing some of the energy from being reflected. Thus, highly transparent graphene is an excellent candidate material for the gate electrode.^38^, ^39^, ^40^ Moreover, an additional advantage of MoS_2_ used in the photodetector is its extremely limited thermal capacity and limited absorption of infrared irradiation. Overall, we assert that the optoelectronic performance of the photodetector can be optimized by developing a method for fabricating a substrate‐free device.

In the 375 nm to 2 µm waveband range, MoS_2_ exhibits excellent photoresponse performance through the modulation of the polarization field, which had been studied by our group.^30^ In present work, improvements to the device structure and fabrication process result in further enhancing the optoelectronic performance in the UV–SIR range. Specifically, the functional material is MoS_2_, and the auxiliary material is P(VDF‐TrFE); roles of the materials are interchanged compared with those in the previous device operation. MoS_2_ produces a photocurrent based on its photoconductive effect, and when the energy of the incident light exceeds that of the bandgap, numerous photogenerated carriers contribute to the photocurrent.^41^ Furthermore, the upward remanent polarization electric field further improves the optoelectronic performance of the device by, for example, suppressing the dark current, shortening the photoresponse time (including both rise and fall times), and improving the on/off photocurrent switching ratio, photoresponsivity, and detectivity. More importantly, the bandgap for MoS_2_ is regulated by the strong local polarization field, yielding a smaller bandgap, and the photoconductive effect of MoS_2_ extends the detection cut‐off wavelength to the SIR region. As indicated in Figure 4d, a series of responsivities and on/off photocurrent switching ratios of the photodetector at *V*_SD_ = 1 V were calculated in the 375 nm to 2 µm spectral range. It can be seen that the device exhibits ultrahigh on/off photocurrent switching ratio beyond 10^4^ under visible light, which mainly depends on the photoconductive effect enhanced by the polarization field. Especially for incident light at 637 nm, the responsivity is 3260 A W^−1^ and detectivity is 9 × 10^14^ Jones when the drain bias is 5 V; the current rise (*t*_r_) and decay (*t*_f_) times are only 480 and 320 µs, respectively (Figure S6, Supporting Information), which are all favorable results compared with our previous work.^30^ Detailed optoelectronic data for wavelengths ranging from 375 nm to 2 µm are provided in Figure S7 (Supporting Information).

On the basis of the excellent optoelectronic performance of the device under visible light irradiation, we conducted a single‐pixel imaging experiment. **Figure** **5**a displays the setup of the single‐pixel imaging technique.^42^ The imaging target is a patterned light source, and the detector is fixed in a camera obscura located on a 2D rotary table. The imaging target is captured by the camera lens and focused on the detector. By controlling the movement of the 2D rotary table, the detector scanned the target line by line. Then, the photocurrent signals of the detector are recorded using a computer; a high‐quality Shanghai Institute of Technical Physics (SITP) image with visible light is achieved, as displayed in Figure 5b.

**Figure 5:**
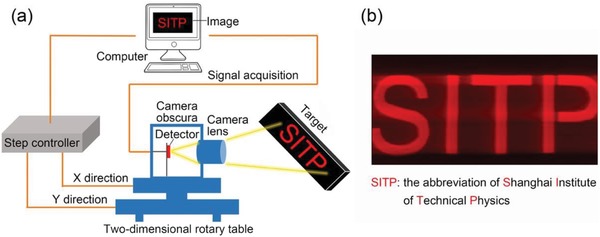
a) Single‐pixel imaging setup. b) Visible light image “SITP” captured using the single‐pixel imaging technique.

In summary, we developed a multimechanism synergistic photodetector exhibiting ultrabroad spectrum response and excellent optoelectrical performance. On the basis of the harmonious cooperation of P(VDF‐TrFE) and MoS_2_, the pyroelectric and photoconductive properties of both materials were greatly improved. In the MIR–LIR range, 2D MoS_2_ is used to read and amplify the photocurrent given rise by the pyroelectric effect of P(VDF‐TrFE), where MoS_2_ is in the depletion state and highly sensitive to the surface charge induced by ferroelectric polarization. Consequently, with infrared irradiation, higher sensitivity of pyroelectric effect is achieved. In the UV–SIR range, the P(VDF‐TrFE) polarization field dramatically improved the optoelectronic performance of MoS_2_, establishing it as highly suitable for visible light imaging. Overall, combining the advantages of MoS_2_ and P(VDF‐TrFE) significantly improves the optoelectronic performance of the photodetector, which is a reliable route to prepare high‐performance room‐temperature ultrabroad spectrum photodetectors.

Looking ahead, combining mechanisms in a single device is an effective and straightforward method that can be used in future multifunctional applications. For example, integrating the piezoelectricity^43^ or flexibility^44^ of P(VDF‐TrFE) with 2D materials (not limited to MoS_2_, but also other 2D semiconductors) is desirable. Our comprehensive study of a 2D photodetector driven by a ferroelectric polymer provides a feasible approach for reforming infrared detectors on the basis of the pyroelectric effect and paves the way for the development of next‐generation multifunctional, power‐efficient, and uncooled optoelectronic applications.

## Experimental Section

For device fabrication, a polyimide film was first prepared. An amic acid solution (Sigma‐Aldrich Trading Co., Ltd.) was spin‐coated at 8000 rpm for 1 min on the SiO_2_/Si substrate and then annealed at 220 °C for 2 h to obtain an ultrathin (≈1.7 µm) polyimide film. Few‐layer MoS_2_ flakes were then mechanically exfoliated from bulk single crystals (HQ Graphene, Inc.) and transferred onto the polyimide film. Bilayer photoresists of LOR‐3A and AZ‐1500 were sequentially spin‐coated (3000 rpm for 20 s) onto the polyimide and then baked in an oven at 90 °C for 30 min. UV lithography was used to define the patterns of the source and drain, which had a channel length fixed at 5 µm. The channel width was ≈4–10 µm for different samples. Thus, the active area of the photodetector was about 20–100 µm^2^, which is the channel area. Cr/Au (15/50 nm) metal films served as the source/drain electrode were deposited by a thermal evaporator before the lift‐off step. The device was then annealed with a temperature of 200 °C and maintained 2 h in a vacuum chamber filled by Ar_2_. Next, a P(VDF‐TrFE) (70%/30% in mol) solution was spin‐coated (3000 rpm for 20 s) as the gate dielectric. After spin‐coated six times, a 300 nm P(VDF‐TrFE) film was yielded; this film was then annealed at a temperature of 135 °C for 4 h in an oven to enhance its crystallinity. Next, through the electron beam evaporation method, an 8 nm Al metal film was fabricated on the top of P(VDF‐TrFE). There will be about 1–2 nm of Al oxidized to Al_2_O_3_, but this will not destroy the polarization of P(VDF‐TrFE). Subsequently, the gate electrode was patterned by UV photolithography (negative photoresist) followed by Ar‐ion etching. The residual photoresist was then removed through O_2_ plasma etching. Finally, the device on the polyimide film was delaminated from the SiO_2_/Si substrate.

Polyimide, MoS_2_, and P(VDF‐TrFE) are the three critical materials that determine the optoelectronic performance of a device. First, the characterization of polyimide is presented in Figure S4a,b (Supporting Information), and the molecular structure of the amic acid solution (C_12_H_12_N_2_O · C_10_H_2_O_6_)*_n_* is depicted in the inset of Figure S4a (Supporting Information). After spin‐coating on the SiO_2_/Si substrate and annealing at 220 °C for 2 h, a step profiler (NanoMap 500LS) was used to measure the thickness of the polyimide film, which was ≈1.7 µm. Second, the thickness of MoS_2_ was confirmed through Raman spectroscopy (Lab Ram HR800) using an excitation wavelength of 532 nm, a power of 2 mW, and a spot size of 2 µm. In this study, MoS_2_ selected as the channel had a few layers (2–5 layers). The optical images of four MoS_2_ samples on the polyimide and their Raman spectroscopy results are displayed in Figure S4c,d (Supporting Information). Third, the thickness of P(VDF‐TrFE) (Figure S4e, Supporting Information) was also measured using the step profiler. Figure S4f (Supporting Information) characterizes the pyroelectricity of 300 nm P(VDF‐TrFE) used in this study. Pyroelectricity of P(VDF‐TrFE) was derived from the temperature dependence of the remanent polarization for a capacitor. The temperature dependence of remanent polarization was evaluated using a ferroelectric analyzer (Precision LC.) on a variable‐temperature probe station (MMR Technologies, Inc.).

All electronic and optoelectronic measurements were carried out at room temperature and under ambient conditions. Electronic measurements were conducted using an Agilent B2902A source measure unit on a Lake Shore probe station. The optoelectronic measurement setup for incident light wavelengths from 375 nm to 2 µm is presented in Figure S8a (Supporting Information). Incident light was provided by a monochrome laser, but any laser wavelength in the 375 nm to 2 µm range can be used. This laser was focused on the object (device) through a series of optical lenses and a microscope. The bulb and CCD camera in the light path were used to precisely control the position of the laser spot ensuring that the laser was incident on MoS_2_. The electrodes of the device were connected to a semiconductor device analyzer (Agilent B2902A) equipped with a transfer box. The optoelectronic signals induced by the incident light were thus captured by the semiconductor device analyzer. The current signal was converted to a voltage signal using a preamp (Stanford Research Systems SR570) and was displayed on an oscilloscope (Tektronix MDO3014). All data were recorded using a computer. Figure S8b (Supporting Information) shows the optoelectronic measurement setup for incident light wavelengths from 2.76 to 10 µm. In this case, the infrared laser exited the laser device and then irradiated on the object (device), and a beam of visible light that had the same pathway as the infrared laser was used to calibrate the position of the infrared laser. At the beginning of the test, this visible light was filtered by a germanium lens. The wavelength of the infrared laser was tuned using the laser controller. As in the aforementioned case, optoelectronic signals were collected using the semiconductor device analyzer. For frequency‐dependent measurements, the current signal was converted to a voltage signal using the preamp. A phase detector (Signal Recovery Model 7270 DSP Lock‐in Amplifier) was used to lock this voltage signal at the same frequency as the chopper. The oscilloscope then displayed the variation induced by the infrared light.

## Conflict of Interest

The authors declare no conflict of interest.

## Supporting information

Supplementary
