# Supplementary material for: Multimechanism Synergistic Photodetectors with Ultrabroad Spectrum Response from 375 nm to 10 µm
Source: Adv Sci (Weinh). 2019 Jun 4;6(15):1901050. doi: 10.1002/advs.201901050 (PMC6685502; doi:10.1002/advs.201901050)
Supplement: Supplementary file 1 — Supplementary [file ADVS-6-1901050-s001.pdf]

Copyright WILEY-VCH Verlag GmbH & Co. KGaA, 69469 Weinheim, Germany, 2019.

ADVANCED SCIENCE
Open Access

Supporting Information

for *Adv. Sci.,* DOI: 10.1002/advs.201901050

Multimechanism Synergistic Photodetectors with Ultrabroad Spectrum Response from 375 nm to 10 µm

Xudong Wang, Hong Shen, Yan Chen, Guangjian Wu, Peng Wang, Hui Xia, Tie Lin, Peng Zhou, Weida Hu, Xiangjian Meng, Junhao Chu, and Jianlu Wang**

Supporting Information for

Multi-mechanism synergistic photodetectors with ultrabroad spectrum response from 375 nm to 10 μm

Xudong Wang[1†], Hong Shen[1†], Yan Chen[1,2†], Guangjian Wu[1], Peng Wang[1], Hui Xia[1], Tie Lin[1], Peng Zhou[3], Weida Hu[1*], Xiangjian Meng[1], Junhao Chu[1], Jianlu Wang[1*]

[1] *State Key Laboratory of Infrared Physics, Shanghai Institute of Technical Physics, Chinese Academy of Sciences, 500 Yu Tian Road, Shanghai 200083, China.*

[2] *University of Chinese Academy of Sciences, 19 Yuquan Road, Beijing 100049, China.*

[3] *Department of Microelectronics, State Key Laboratory of ASIC and System, Fudan University, Shanghai 200433, China.*

[*] Corresponding authors. Email: wdhu@mail.sitp.ac.cn; jlwang@mail.sitp.ac.cn;

[†] These authors contributed equally to this work.

Figure S1. Device optical images.

Figure S2. Electrical characteristics of the device and P(VDF-TrFE).

Figure S3. Optoelectronic performance of a P(VDF-TrFE)-based pyroelectric detector.

Figure S4. Characterizations of the polyimide, few-layer MoS_2, and P(VDF-TrFE).

Figure S5. Photocurrent switching with incident light in the MIR−LIR range.

Figure S6. Optoelectronic performance with 637 nm incident light and ferroelectric polarization field effects on photoluminescence spectrum of MoS_2.

Figure S7. Photocurrent switching with incident light in the UV−SIR range.

Figure S8. Optoelectronic measurement setups.

Note S1. Calculation of the normalized energy flux profile of the device.

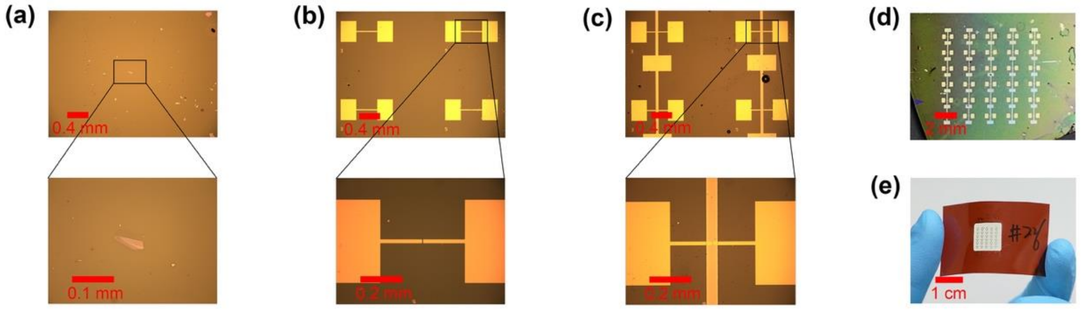

Figure S1. a) Micrographs of few-layer MoS_2 on the polyimide substrate captured at magnifications of 50× (top panel) and 500× (bottom panel). b) Micrographs of few-layer MoS_2 with source/drain electrodes captured at a magnification of 50× (top panel) and 200× (bottom panel). c) Micrographs of the device after fabricating the top gate, which were captured at magnifications of 50× (top panel) and 200× (bottom panel). d) Photograph of the device before separation from Si/SiO_2. e) Photograph of the device after delamination from Si/SiO_2 (#226 is the identification number of the device used for the experiment).

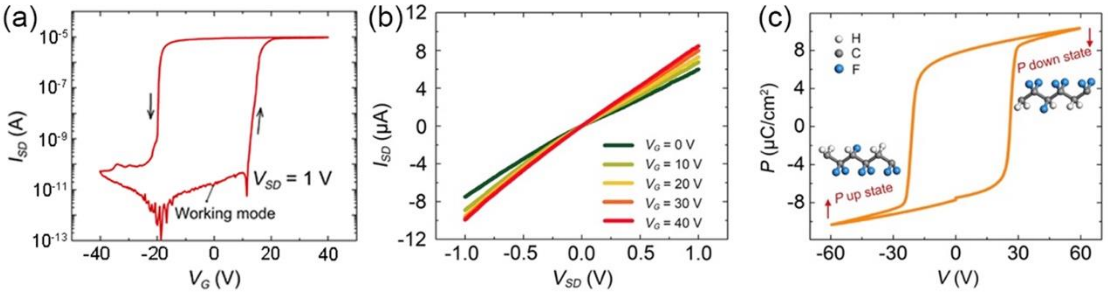

Figure S2. a) Transfer curve of the MoS_2 FET gated by P(VDF-TrFE) at a drain bias of 1 V. The large counterclockwise hysteresis window in this curve is attributed to the ferroelectricity in P(VDF-TrFE). In the upward polarization state of P(VDF-TrFE), the drain current can be maintained at an ultralow level, which is selected as the working mode. Field-effect mobility (μ) was estimated to be approximately 75.3 $cm^2V^{-1}s^{-1}$. b) Output curves of the device acquired for different gate voltages and P(VDF-TrFE) has been polarized downward. c) The hysteresis loop for 300 nm P(VDF-TrFE). Inset: molecular structure schematics for P(VDF-TrFE) with downward polarization (right panel) and upward polarization (left panel) states.

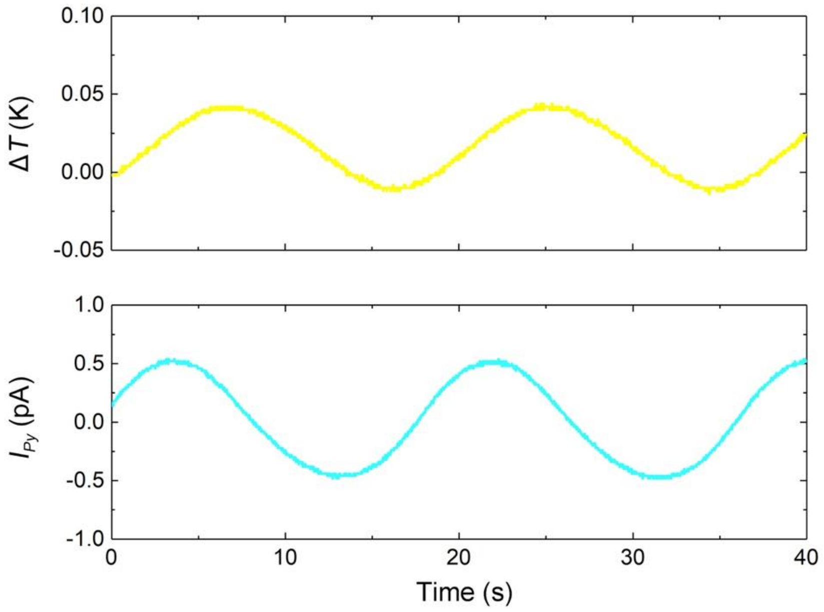

Figure S3. The pyroelectric current (I_{Py}) of a P(VDF-TrFE)-based pyroelectric detector was measured under sinusoidal infrared irradiation, which was converted into a temperature variable (ΔT) by measuring the resistance of a platinum flake. The pyroelectric current of the device caused by infrared irradiation was only a few picoamperes, which must be amplified for favorable detector performance. This P(VDF-TrFE) capacitor was fabricated on a silicon substrate which thickness is approximately 500 μm. Most of the heat induced by infrared irradiation is absorbed by the silicon substrate, resulting in an about 1.5 s delay between pyroelectric current and infrared irradiation.

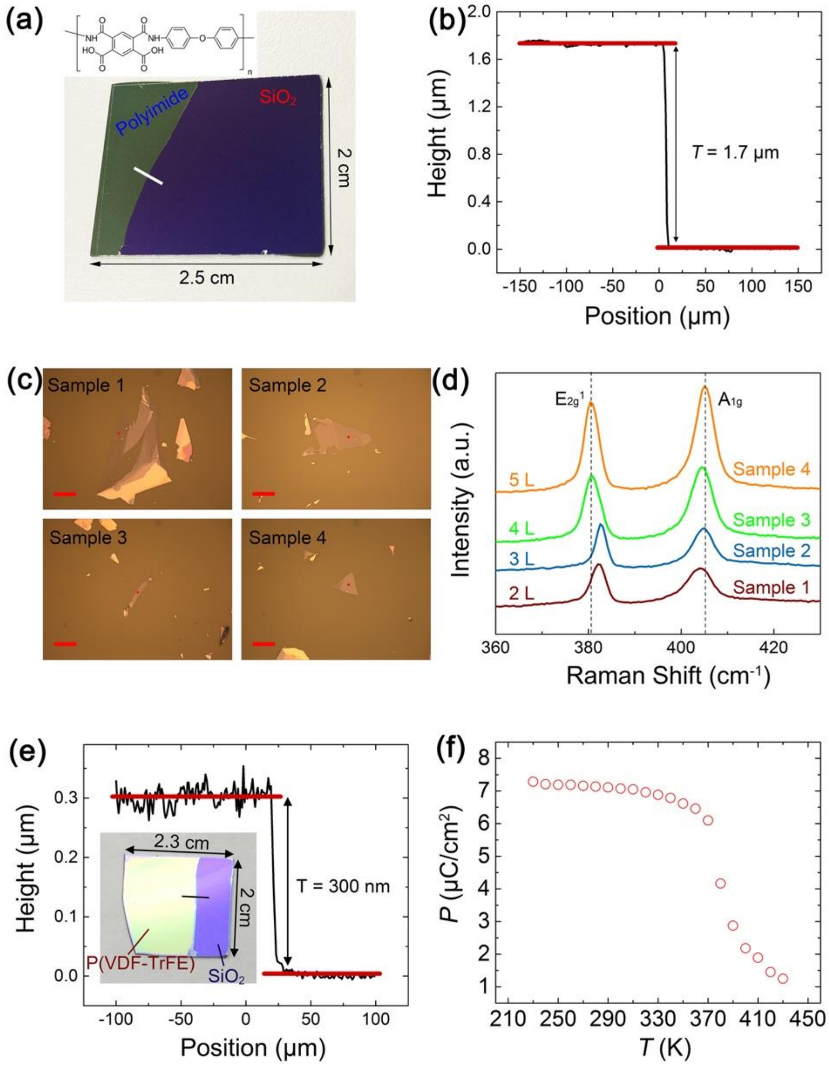

Figure S4. a) Photograph of the polyimide on SiO_2/Si; the inset displays the molecular structure of the amic acid solution. b) Thickness of the polyimide, measured using a step profiler along the write line in (a). c) Optical images of the four MoS_2 samples with few-layers used to prepare the devices in this study. All scale bars are 20 μm, and the red dot in each image denotes the Raman test zone. d) Raman spectra of the MoS_2 samples in (c). The thickness of MoS_2 was determined according to the difference in peak position between E_{2g}^1 and A_{1g}. e) The thickness of P(VDF-TrFE) was approximately 300 nm along the black line in the inset. The inset depicts the sample used to measure P(VDF-TrFE) thickness. f) Relationship between the remanent polarization of P(VDF-TrFE) and temperature. Remanent polarization decreased significantly when the temperature exceeded above 370 K.

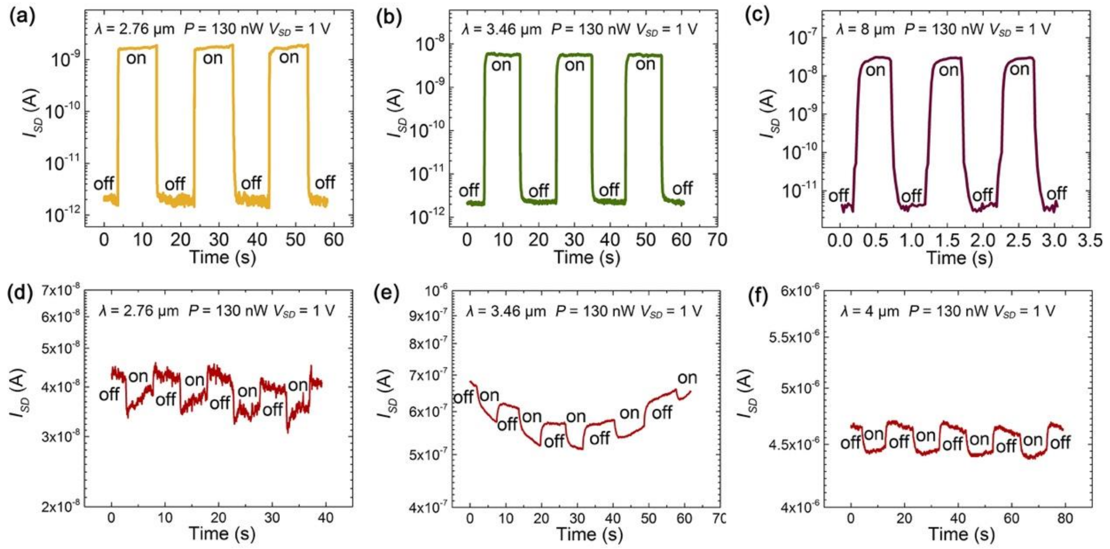

Figure S5. a–c) Photocurrent switching behaviors of P(VDF-TrFE) in the P_{up} state with incident light wavelengths of 2.76, 3.46, and 8 μm. When the device was heated by infrared light, the positions of aligned atoms in the ferroelectric domain changed immediately. Consequently, the upward remanent polarization of P(VDF-TrFE) reduced and weakened the effect of field modification on MoS_2; subsequently, the number of electrons in the MoS_2 channel increased, and the device showed sensitivity to photocurrent switching. d–f) Photocurrent switching behaviors of P(VDF-TrFE) in the P_{down} state with incident light wavelengths of 2.76, 3.46, and 4 μm. In this case, the number of electrons in the MoS_2 channel decreased with infrared light and the downward polarization of P(VDF-TrFE). This behavior was similar to the negative photocurrent switching effect. All measurements were performed under the same incident laser intensity (P = 130 nW) at V_{SD} = 1 V.

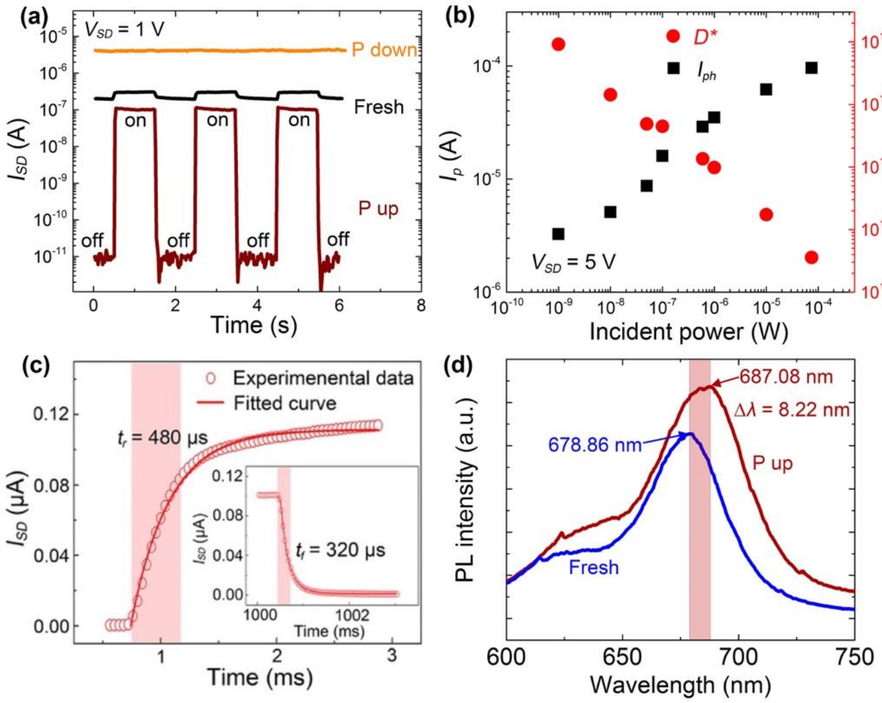

Figure S6. a) Photo-switching behaviour under three states: fresh, P_{up}, and P_{down} (λ = 637 nm, P = 100 nW). b) Incident power dependence according to photocurrent and detectivity (λ = 637 nm, V_{SD} = 5 V). The maximum $R \approx 3260$ A/W and $D^* \approx 9 \times 10^{14}$ Jones were achieved when the incident power was 1 nW. c) Photoresponse time (rise and fall) of the photodetector with 637 nm laser incidence, which was fitted by exponential functions. The current rise (t_r) and decay (t_f) times were only 480 μs and 320 μs, respectively. d) Photoluminescence spectrums of MoS_2 under the fresh state (blue line) and P_{up} state (red line). The photoluminescence spectrum of the sample in the P_{up} state showed an apparent redshift compared with that of the fresh sample. The difference between the peaks was approximately 8.22 nm.

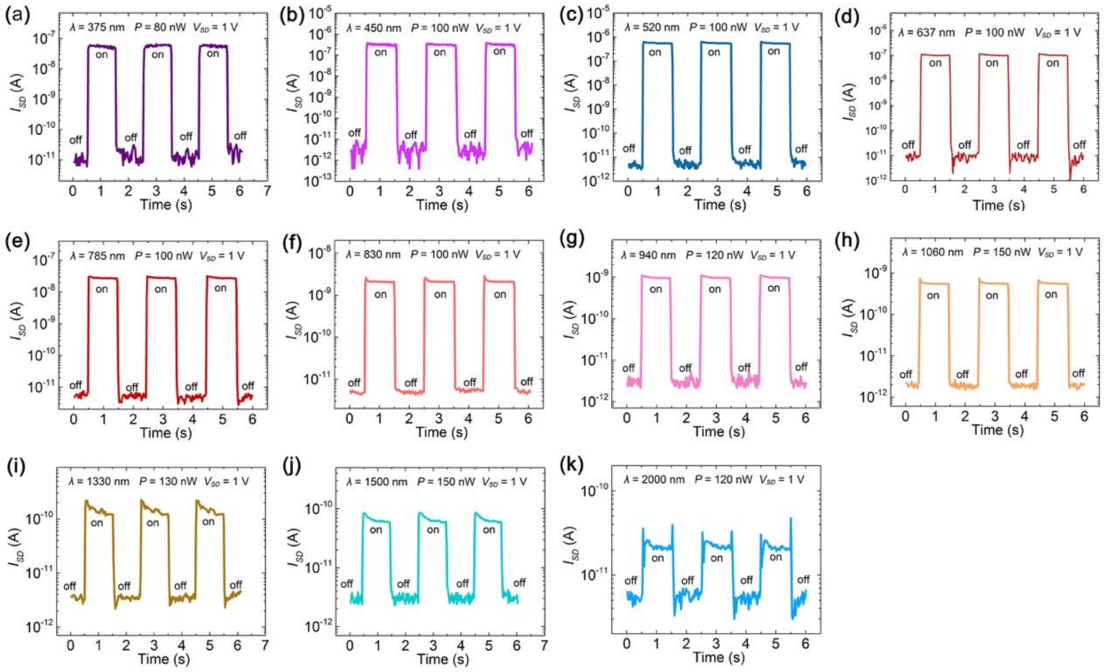

Figure S7. a−k) All measurements were performed under the same drain bias (V_{SD} = 1 V) with different incident light wavelengths and powers. The on/off photocurrent switching ratio (I_{ph}/I_{dark}) was easily obtained for each laser incident wavelength. As the wavelength of incident light increases, the incident photon energy decreases, resulting in a reduced photocurrent and photocurrent switching on/off ratio.

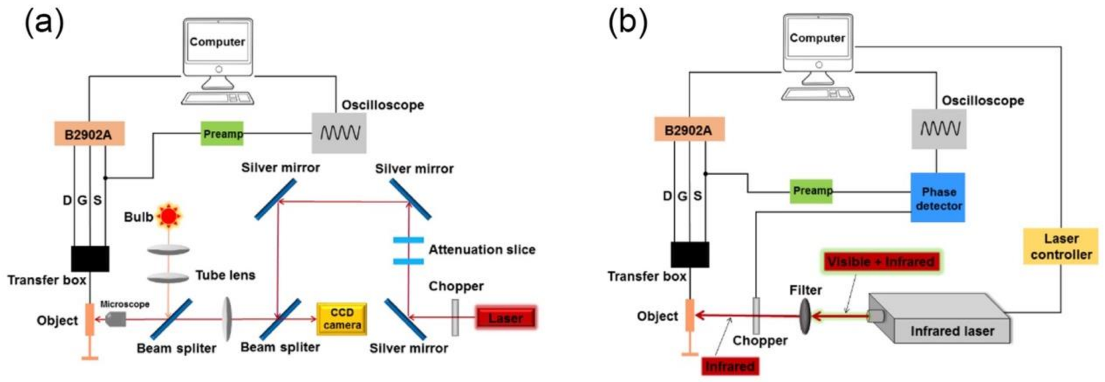

Figure S8. a) Optoelectronic measurement setup for incident light wavelengths from 375 nm to 2 μm. b) Optoelectronic measurement setup for incident light wavelengths

from 2.76 μm to 10 μm.

Note S1. Calculation of the normalized energy flux profile

The normalized energy flux (S^*) profile of the stacked layers was calculated using the transfer-matrix method. We assumed that the stacked layers were normalized to the Z-axis, and that infrared radiation was normally incident on the stacked layers. Accordingly, the polarization of infrared radiation was in the X–Y plane and was therefore neglected.

The electric field within one layer is represented as

$$E(z) = E_+ \exp(ikz) + E_- \exp(-ikz) \tag{1}$$

where E is the electric field, k is the wave vector, and E_+ and E_- are the coefficients of the forward-traveling and backward-traveling waves, respectively. The corresponding magnet field can be calculated as

$$\nabla \times \vec{E} = -\frac{\partial \vec{B}}{\partial t} \Rightarrow -i\omega B(z) = ik[E_+ \exp(ikz) - E_- \exp(-ikz)]$$
$$\Rightarrow cB(z) = \mathrm{n}[E_+ \exp(ikz) - E_- \exp(-ikz)]$$

(2)

where B is the magnet field, ω is the angular frequency, c is the speed of light in a vacuum, and n the complex refractive index. According to the Maxwell equation, E and B are continuous not only in each layer but also at the interface; therefore, we constructed a vector

$$V \equiv \begin{pmatrix} V_1 \\ V_2 \end{pmatrix} \equiv \begin{pmatrix} E \\ icB \end{pmatrix} \tag{3}$$

which is continuous in the stacked layers. Combining Eq. (1)–(3), a transfer matrix of one layer, M, is defined as

$$V_e = MV_i$$
$$M = \begin{pmatrix} \cos\delta & \sin\delta / n \\ -n\sin\delta & \cos\delta \end{pmatrix} \quad (4)$$
$$\delta = kd = 2\pi d / \lambda$$

where V_i (V_e) is the vector at the wave-incident (ejection) boundary of the layer, λ is the wavelength of the infrared radiation in a vacuum, and d is the thickness of the layer. Taking r (and t) as the amplitude reflectance (and transmittance) coefficients of the stacked layers, we obtained

$$\begin{pmatrix} t \\ 0 \end{pmatrix} = M_n M_{n-1} \cdots M_2 M_1 \begin{pmatrix} 1+r \\ i(1-r) \end{pmatrix} \quad (5)$$

where M_j (j = 1, ..., n) is the transfer matrix of each layer; and the amplitude of the incident wave was normalized to 1. By solving Eq. (5), we could obtain r and t and sequentially the vectors at each interface. The vector inside a layer could be calculated using Eq. (4) by replacing d with the desired distance to obtain the vector profile in the stacked layers. Energy flux is given by

$$\bar{\vec{S}} = \frac{1}{2}\mathrm{Re}\left(\vec{E}^* \times \vec{H}\right) \Rightarrow \bar{S} \propto \mathrm{Im}\left(E^*(icB)\right) = \mathrm{Im}\left(V_1^* V_2\right) \quad (6)$$

where the last term is the normalized energy flux and can be obtained using the vector profile.
